# Do positive childhood experiences reduce children’s deviant behavior in physical education learning?—A longitudinal validation based on a latent variable growth model

**DOI:** 10.3389/fpsyg.2026.1920476

**Published:** 2026-09-14

**Authors:** Yuyang Hou, Wangang Lv, Ye Bi, Menglin Zhang

**Affiliations:** 1 School of Physical Education, Wuhan Sports University, Wuhan, China; 2 Office of International Affairs, Wuhan Sports University, Wuhan, China; 3 Economy & Management School, Wuhan Sports University, Wuhan, China

**Keywords:** child educational psychology, children’s physical education, children’s wellbeing, deviant behavior, life experiences

## Abstract

In recent years, there has been a global decline in children’s interest in participating in physical education classes, a phenomenon that has garnered continuous attention in the fields of physical education and sports psychology yet lacks systematic micro-level exploration and analysis. This study employed a questionnaire survey to conduct three waves of longitudinal testing over 3 years with 284 Chinese children. Based on cognitive behavioral theory, a theoretical model was constructed, and latent variable growth modeling and cross-lagged regression were used to analyze the trends in positive childhood experiences and children’s deviant behavior in physical education learning, exploring the relationship between the development of positive childhood experiences and the development of children’s deviant behavior in physical education learning, as well as the temporal effects between positive childhood experiences and children’s deviant behavior in physical education learning. The results revealed that: (1) the impact of positive childhood experiences on children showed a linear increasing trend with age, while children’s deviant behavior in physical education learning showed a linear decreasing trend with age; (2) the initial level of positive childhood experiences negatively predicted the initial level (β = −0.255, *p* < 0.05) and the growth rate (β = −0.346, *p* < 0.05) of children’s deviant behavior in physical education learning; (3) the rate of decrease in positive childhood experiences significantly predicted the growth rate of children’s deviant behavior in physical education learning (β = −0.429, *p* < 0.05); (4) the impact of positive childhood experiences on children significantly negatively predicted deviant behavior in physical education learning 1 year later (βt1 = −0.152, *p* < 0.05; βt2 = −0.244, *p* < 0.05). This paper aims to provide theoretical references and some practical insights for preventing and addressing various potential issues in children’s physical education teaching.

## 1 Introduction

The sudden outbreak of the COVID-19 pandemic in 2019 plunged people worldwide into immense panic (Trougakos et al., 2020). Particularly in the education sector, which is closer to the public, the pandemic not only caused passive disruption to global education order but also increased the economic burden on schools and education departments while making it difficult to ensure teaching quality, significantly affecting the physical and mental health of teachers and students (Franic and Dodig-Curkovic, 2020). Influenced by contemporary consumerism, most media focus on building and promoting a healthy body, and the enhancement of immunity has gradually become a primary concern in the health field of the current era (Gombart et al., 2020). Active participation in physical activities and the development of sports habits are widely regarded as an economical and effective way to strengthen the body (da Silveira et al., 2021). However, despite strong advocacy from governments and commercial media, most individuals today have not developed the good habit of regularly participating in physical activities (Guthold et al., 2020; Strain et al., 2024). The reasons for this are, on one hand, that the cultivation of sports habits cannot be achieved overnight, and on the other hand, school physical education remains a relatively weak link in global education (Strain et al., 2024). Today, children like sports but do not like physical education classes, making it difficult for them to develop a stable interest in sports during their growth and affecting their lifelong exercise interest.

Primary school physical education classes, as the first barrier for children to systematically learn sports activities, play an extremely important enlightening role in fostering children’s future sports participation and sports habits (Keegan et al., 2019). However, in Chinese primary schools, it has become common for physical education classes to be occupied by subjects such as mathematics, or for physical education classes to be conducted online through video viewing. This leads to a lack of collective physical exercise time for children, no time for sports activities, resulting in a loss of interest in sports, and then to poor physical fitness, subsequently leading to active avoidance of exercise, forming a vicious cycle. Besides these objective reasons, deviant behavior is the most significant internal cause of inefficient learning in children’s physical education classes (Baños et al., 2024). Physical education learning is a process that promotes children’s physical and mental development and integrates moral education into physical activities. However, some children still lack awareness of classroom norms and often exhibit deviant behaviors in physical education learning, such as slackness, laziness, tardiness, academic weariness, skipping classes, dishonesty, non-cooperation, and hostility toward others, resulting in children failing to develop a good learning attitude after years of physical education learning. This deviant behavior has become a common bad behavior in physical education learning, which not only affects children’s learning outcomes but also has irreversible negative effects on their future sports participation.

But from the existing research literature, studies on children’s deviant behavior in physical education learning are extremely scarce. Existing studies generally equate children’s deviant behavior in physical education classes with children’s problem behavior in physical education classes. Research on deviant behavior in physical education learning mainly focuses on two disciplines: pedagogy and psychology (Hovdal et al., 2021). From the perspective of physical education, PE classes are characterized by experiential and embodied learning, and children’s passive coping behaviors or conflicts with peers are often directly embedded in specific movement tasks. Existing research in physical education has focused predominantly on motor-skill development and participation motivation, whereas comparatively limited attention has been paid to the developmental trajectories of such behaviors and the experiential foundations rooted in children’s everyday lives. Specifically, most existing related literature concentrates on the causes and manifestations of classroom behavior problems. Although such studies provide information on the development of children’s deviant behavior in learning, they mostly rely on cross-sectional data, making it difficult to reveal the true situation of individual development (Keresztes et al., 2022). Moreover, although a growing body of research has examined children’s deviant behavior in learning, comparatively limited attention has been devoted to its developmental trajectory across time. Longitudinal evidence concerning deviant behavior specifically within physical education learning also remains relatively scarce. Consequently, the ways in which such behavior changes during childhood, as well as the contextual factors associated with these changes, have not yet been fully clarified.

It is important to recognize that physical education is not an ordinary academic setting. Differences in body shape and physical fitness are often made visible under the gaze of peers, while teachers’ demonstrations of motor skills and assessments of physical performance render evaluation comparatively public. For children whose physical self-concept has not yet stabilized, such visible displays of performance may readily be experienced as social comparison and scrutiny, thereby eliciting negative emotional responses. Previous research has indicated that concerns about negative evaluation of physical performance may affect children’s self-esteem and social interactions, whereas positive life and sport experiences may help alleviate such anxiety (Liu et al., 2025). From this perspective, positive childhood experiences may provide a developmentally meaningful point of departure for understanding children’s deviant behavior in physical education learning. Positive childhood experiences refer not merely to the absence of adversity, but also to the support, acceptance, sense of belonging, and experiences of success that children obtain through interactions with family members, schools, and peers. These experiences may foster psychological safety and a sense of self-worth, enabling children to interpret mistakes made in physical education as improvable learning events rather than as global reflections of personal inadequacy. Previous studies have shown that higher levels of positive childhood experiences are associated with lower levels of anxiety, depression, and behavioral problems among children and may attenuate the association between adversity and psychological difficulties (Lyons and Suh, 2025). Among early adolescents, such experiences have also been associated with lower levels of aggression and rule-breaking behavior (Logan and Lewis-de Los Angeles, 2025). Accordingly, positive life experiences and supportive experiences in physical activity may reduce children’s need to protect themselves by disrupting classroom order or resisting the rules of physical activity, partly by buffering evaluation-related anxiety in physical education settings. In turn, these experiences may help inhibit the emergence and accumulation of deviant behavior in physical education learning.

Based on this, to enhance the understanding of children’s deviant behavior in physical education learning, this study, grounded in cognitive behavioral theory, introduces the variable of positive childhood experiences, which is closely related to children’s life, learning, and growth. Using longitudinal data and applying unconditional latent variable growth modeling and cross-lagged regression models, we examine the developmental trajectories of positive childhood experiences and children’s deviant behavior in physical education learning and the causal relationship between them. This study aims to help educators accurately identify key positive childhood experience factors that influence children’s deviant behavior in physical education learning, thereby providing a scientific basis for developing targeted intervention measures.

## 2 Literature review

### 2.1 Deviant behavior in physical education learning

Deviant behavior is also defined as “problem behavior,” “misbehavior,” or “delinquent behavior” (Bandura et al., 1996). Children’s deviant behavior refers to conduct exhibited by children during interactions with others that does not conform to social norms in terms of character and morality (Kazdin, 1980). In the educational context, learning deviant behavior is regarded as a common type of problem behavior that violates school rules and discipline and deviates from moral standards. Consequently, children’s learning deviant behavior is defined as behavior that violates and deviates from social moral norms, generated during children’s growth due to the influence of both internal and external factors, which can severely hinder children’s healthy development (Finlay-Jones et al., 2024).

Regarding the manifestations of children’s learning deviant behavior, scholars have found that some children exhibit a series of behavioral problems and psychological disorders from an early age (Blok et al., 2022). Concerning the causes, scholars generally agree that the origins of deviant behavior are multifaceted, involving individual, family, school, and societal levels (Oh et al., 2024). However, the academic community consistently recognizes life environment and family environment as significant factors influencing children’s learning deviant behavior (Nīmante, 2023). Children’s physical education learning is an important pathway for cultivating physical fitness, teamwork, and positive attitudes (Carson et al., 2016). Nevertheless, some children display deviant behaviors such as hitting peers or intentional absenteeism during physical education learning. This not only affects children’s learning outcomes in physical education and constrains their interest in it but also becomes a major obstacle to promoting the concept of “lifelong sports.” Summarizing existing research indicates that the primary cause of such deviant behavior in children lies in the family environment. The family environment exerts a tremendous influence on children’s physical education learning. Additionally, relationships with classmates are also an important influencing factor (Li et al., 2025). Social problems may make children feel isolated in physical education classes, increasing the occurrence of deviant behavior. Finally, the teaching attitude of physical education teachers is crucial for children’s interest in physical education learning; teachers who are overly strict or indifferent may provoke aversion and resistance in children.

In research concerning children’s deviant behavior in physical education learning, this paper finds that children’s performance behaviors in physical education classes differ across age groups (Bélanger et al., 2015). Among these, physiological differences are a main factor for deviant behavior in lower-grade children (Grades 1–3), who are more likely to feel frustrated with physical activities due to relatively poorer physical abilities and thus exhibit deviant behavior. Older children, around 11 years of age, are in the transition stage between school age and adolescence, characterized by lower learning concentration, while their families often have higher expectations. These expectations may lead to excessive competition in physical education learning, resulting in deviant behavior (Zhong and Wang, 2025). In summary, deviant behavior in children’s physical education learning is a complex, multi-faceted issue. Children of different ages exhibit distinct characteristics and influencing factors regarding such behavior.

### 2.2 Positive childhood experiences

Currently, definitions of positive childhood experiences vary among scholars. Most typically define its core connotation from the perspectives of psychological and emotional support, focusing primarily on the emotional care, attention, and encouragement individuals receive during childhood. This includes positive feedback, love, understanding, and affirmation of children’s self-esteem and self-worth from parents or other caregivers. For instance, some scholars define positive childhood experiences as experiences where individuals felt cared for, loved, protected, and valued during their childhood (Bethell et al., 2019).

Positive childhood experiences have a profound impact on the long-term development of the individual child (Huang et al., 2023). Reviewing existing research, in-depth exploration of positive childhood experiences mainly concentrates on the following three aspects: First, research on its effects on children’s mental and physical health. For example, the findings of Cunha et al. indicate that adolescents who recall warmer and more secure emotional memories of their caregivers from childhood tend to have lower levels of negative emotion (Cunha et al., 2014). Second, research on the development and evaluation of intervention measures. For instance, some studies suggest that positive childhood experiences can offset the long-term effects of childhood negative psychology (Gunay-Oge et al., 2020). Third, the application of positive childhood experiences in positive psychology and their long-term effects on individual growth. For example, some scholars state in their research that positive childhood memories contribute to the generation and expression of positive affect in individuals. Simultaneously, in safe and supportive environments, children can learn to trust others, experience more pro-social interactions, and significantly reduce the occurrence of deviant behavior (Pastorelli et al., 2021). It is undeniable that the influence of positive childhood experiences on child development changes gradually with age stages, and its impact also shows distinct longitudinal variations. When children enter school age, positive childhood experiences begin to significantly affect academic performance (Yang et al., 2019).

Survey research shows that children aged 7–8 in a growth environment demonstrate more positive learning interest and better academic achievement. Upon reaching ages 8–10, children at this stage begin to interact more with peers and learn skills such as cooperation through these interactions and higher participation in subjects like physical education (Kuutti et al., 2022). Regarding physical activity participation. Overall, positive childhood experiences play a key role at different stages of child development, from promoting learning performance to enhancing social skills, and to increasing enthusiasm for activity participation. These experiences lay a solid foundation for the holistic development of children.

### 2.3 Positive childhood experiences and children’s deviant behavior in physical education learning

Previous research has pointed out that psychological deviation is an important influencing factor in the occurrence of children’s deviant behavior (Hsu et al., 2017). Children’s deviant behavior arises from psychological infringement; the negative impact on children’s psychology leads to cognitive biases, which are then reflected in their behavior (Schmidt and Vereenooghe, 2021). To alleviate negative psychological factors in children’s cognition, positive childhood experiences play a significant role. For instance, some scholars have found that positive childhood experiences have a significant negative predictive relationship with emotional instability (Almeida et al., 2023), and some experts point out that students with higher levels of positive childhood experiences are less likely to experience psychological distress such as uncertainty stress, depressive symptoms, and suicidal tendencies (Johnson et al., 2022). Therefore, the mitigating effect of positive childhood experiences on children’s negative psychology is acknowledged, which further indicates that positive childhood experiences provide children with a sound cognitive process.

Cognitive behavior theory (CBT) is composed of cognitive theory and behavior theory. This theory posits that human emotions and behaviors are largely determined by how they construct their world. Initially, cognitive behavior theory primarily provided methodological guidance for mental illnesses and has shown positive effects in treating psychological disorders in children and adolescents (MacGill, 2018), such as depression (Ribeiro et al., 2013). Cognitive behavior theory emphasizes that cognitive processes determine the generation of behavior, and behavior also influences changes in cognition. It suggests modifying cognitions to change beliefs, thereby correcting unreasonable emotions and behaviors (Thoma et al., 2015). Regarding cognition, its formation process involves children making fixed behavioral responses to external changes. Incorrect beliefs or emotions formed during this process can lead to deviant behavior. However, under the influence of the surrounding atmosphere or experienced events, when children develop correct cognition, it can correct or prevent their deviant behavior (Matthys and Schutter, 2023). Therefore, a common characteristic can be extracted from scholars’ research: positive childhood experiences implicitly assist children in reducing psychological issues such as negative emotions and concepts and establish sound cognition. From the perspective of cognitive behavior theory, children with richer and more positive childhood experiences can develop better cognitive processes. During this process, children’s positive cognitive thinking reduces or corrects their irrational emotions and deviant behavior. Thus, this paper posits a connection between children’s positive childhood experiences and deviant behavior in physical education learning; an increase in positive childhood experiences can mitigate the occurrence of deviant behavior in physical education learning.

It should be emphasized that cognitive behavior theory also highlights the expansion and modification of cognitive schemas through learning. Accordingly, positive childhood experiences should not be viewed merely as a protective background for child development; they also constitute contextual resources through which cognitive restructuring and social-emotional competencies can be fostered. Acceptance and support from family members, teachers, and peers provide children with concrete evidence for revising negative automatic thoughts related to physical education. Such experiences may gradually help them challenge cognitive distortions such as “making a mistake will embarrass me” or “a physical education teacher’s evaluation means that I am inadequate,” and reinterpret failure in physical education as a temporary and improvable part of the learning process. Recent research has shown that cognitive behavioral interventions incorporating cognitive restructuring can reduce anxiety and improve negative cognitions and self-perceptions among children, while greater exposure to positive life experiences is associated with fewer severe emotional problems (Alemdar and Karaca, 2025).

At the same time, social and emotional learning is not simply the transmission of knowledge detached from children’s everyday contexts; rather, it is repeatedly acquired and consolidated through positive interactions. Through cooperation, attentive listening, constructive feedback, and effective conflict resolution embedded in positive childhood experiences, children may gradually develop capacities such as emotion regulation and relationship maintenance. When transferred to physical education settings, these competencies may enable children to respond to movement-related mistakes and participation challenges through communication, help-seeking, and sustained engagement, rather than passive coping, aggression, or disruptive behavior. Related studies have also indicated that school-based social and emotional learning programs, including interventions embedded in primary school physical education, can promote learning adjustment and social-emotional competencies (Ha et al., 2025). Thus, cognitive restructuring primarily reduces children’s threatening interpretations of physical education situations, whereas social and emotional learning helps translate adaptive cognitions into actionable forms of self-management and interpersonal behavior. As positive childhood experiences accumulate, these two pathways may mutually reinforce one another, thereby exerting a sustained inhibitory effect on deviant behavior in physical education learning. In summary, this paper proposes the following hypotheses (the theoretical model of this study is shown in Figure 1):

*H1:* The impact of positive childhood experiences on children shows a linear increasing trend over time.

*H2*: Children’s deviant behavior in physical education learning shows a linear decreasing trend over time.

*H3*: The initial level of the impact of positive childhood experiences on children negatively predicts the initial level of children’s deviant behavior in physical education learning.

*H4*: The initial level of the impact of positive childhood experiences on children negatively predicts the developmental change in children’s deviant behavior in physical education learning.

*H5*: The developmental change in the impact of positive childhood experiences on children significantly negatively predicts the developmental change in children’s deviant behavior in physical education learning.

*H6*: Positive childhood experiences negatively predict children’s deviant behavior in physical education learning 1 year later.

**FIGURE 1:**
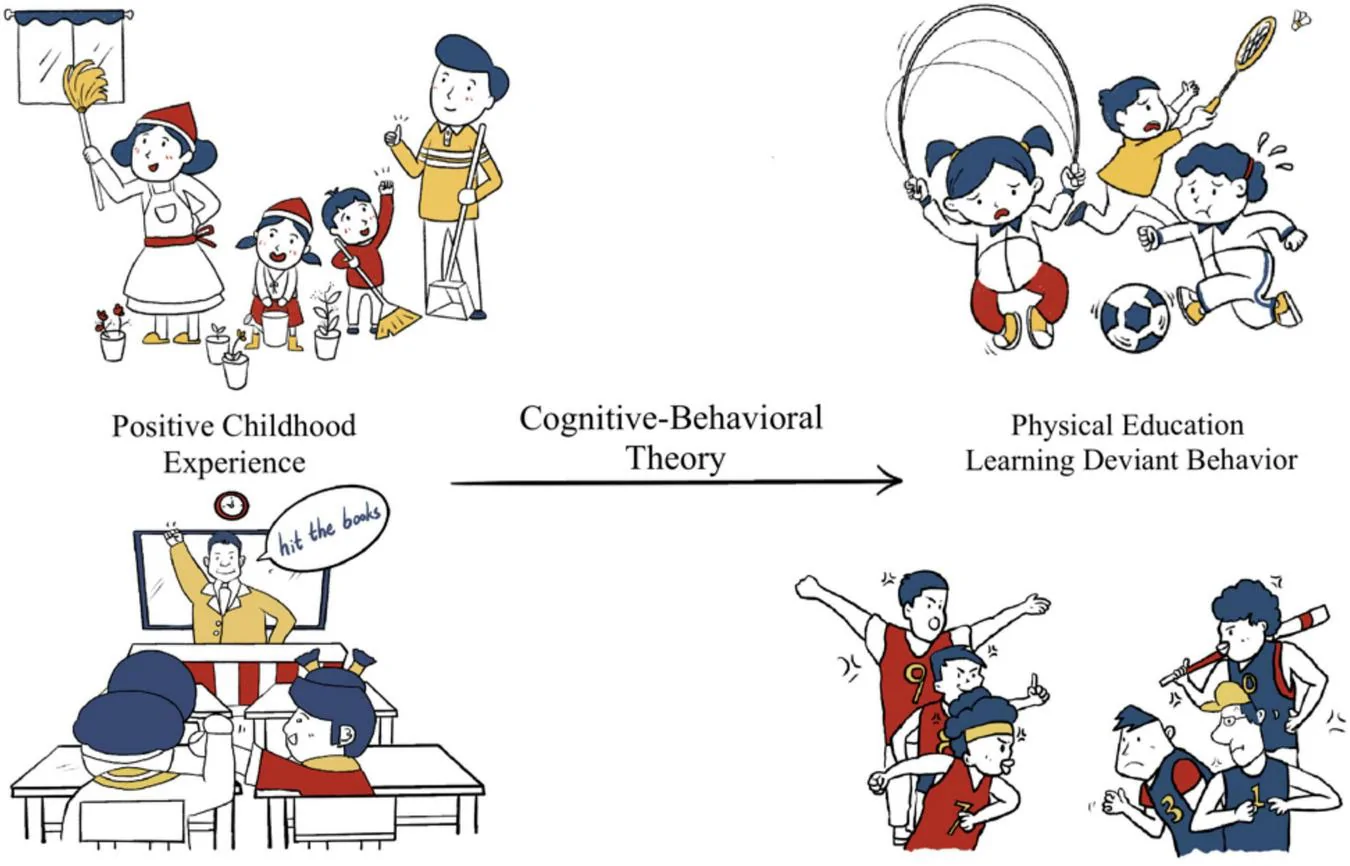
Theoretical model.

## 3 Materials and methods

### 3.1 Participants

This paper is a longitudinal study on the development of children’s physical education and has passed strict ethical review (ethics approval number: 2025156). The studies involving human participants were reviewed and approved by Wuhan Sports University Medical Ethics Committee. Written informed consent to participate in this study was provided by the participants legal guardian. The specific participants were all third-grade students from five primary schools in Western China. This study spanned 3 years, with a total of three testing waves. The first wave was scheduled 1 week after the midterm exams of the third grade. First, teachers were entrusted to distribute the “Parental Informed Consent Form” introducing this study. After parents understood and signed the form, it was collected by the homeroom teacher and handed over to the researchers. Nearly all children’s parents agreed to their children’s participation in this survey. The subsequent two testing waves were conducted 1 week after the midterm exams of the fourth and fifth grades, respectively. The first wave obtained 713 valid participants, the second wave (T2) obtained 411 valid participants, and the third wave (T3) obtained 284 valid participants, including 139 boys and 145 girls. Detailed participant information is shown in Table 1.

**TABLE 1:** Participant Background Information.

Topic	Name	Retained sample (*n* = 284)	Attrited sample (*n* = 429)	χ ^2^/t	*p*
Gender	Male	139 (48.94%)	229 (53.38%)	1.347	0.246
	Female	145 (51.06%)	200 (46.62%)		
Only child status	Yes	155 (54.58%)	229 (53.38%)	0.099	0.754
	No	129 (45.42%)	200 (46.62%)		
Positive childhood experiences T1		3.06 ± 0.84	2.95 ± 0.87	1.591	0.112
Deviant behavior in PE learning T1		3.43 ± 1.12	3.52 ± 0.84	−1.213	0.226

*p < 0.05, **p < 0.01, ***p < 0.001 (the same below).

Considering the importance of sample size in longitudinal studies, this paper analyzed the attrition rate. Specifically, the participant attrition rate for the first wave of data was 42.4%, and for the second wave, it was 30.9%. Based on this, difference tests were conducted between the attrited participants and the retained participants on demographic information, scores of children’s positive childhood experiences at T1, and scores of children’s deviant behavior in physical education learning at T1. First, the data for both attrited and retained participants on demographic information, T1 children’s deviant behavior in physical education learning, and positive childhood experiences showed a certain degree of skewness. Although the data were not normally distributed, the kurtosis of the variables was < 3 and the skewness was < 10, thus they were basically acceptable as normal distributions. Meanwhile, the results of χ^2^ tests and *t*-tests indicated that there were no significant differences between the attrited participants and those who completed all three waves in terms of gender (χ^2^ = 1.347, *P* = 0.246) or being an only child (χ^2^ = 0.099, *P* = 0.754). There were also no significant differences in the scores of positive childhood experiences (*t* = 1.591, *P* = 0.112) and deviant behavior in physical education learning (*t* = −1.213, *P* = 0.226) at T1, indicating that there was no structured attrition in the participants of this study. To examine potential sample selection bias, we further conducted a binary logistic regression analysis. Attrition status was specified as the dependent variable, baseline deviant behavior in physical education learning as the focal predictor, and gender and only-child status as covariates. The effect of baseline deviant behavior in physical education learning was not statistically significant, thereby alleviating, to some extent, concerns that children with more severe deviant behavior in physical education learning were more likely to be lost to follow-up.

### 3.2 Research instruments

The measurement of children’s deviant behavior in physical education learning in this study primarily drew upon the theoretical perspectives of Achenbach and Edelbrock (Achenbach and Edelbrock, 1981), a scale known for its high authority. Based on the characteristics of the research subjects and content, the scale items were simplified, and the wording was appropriately modified to make them easier for children to understand. Following the opinions of several experts, certain items were removed, resulting in the final formal scale. Several scholars in school physical education unanimously agreed that the connotation of this scale aligns with the theoretical conception of deviant behavior in physical education learning. The final scale for children’s deviant behavior in physical education learning consisted of 5 items (including 1 reverse-scored item), such as “I have intentionally been late for physical education class.” The Cronbach’s α coefficients for the three measurements (T1, T2, T3) of deviant behavior in physical education learning were 0.949, 0.949, and 0.932, respectively, all exceeding 0.9, indicating high reliability and stability of the data.

Correspondingly, the measurement of children’s positive childhood experiences in this study primarily utilized the mature scale developed by Zhan et al. (2021) related to children’s positive childhood experiences. Regarding specific dimensions and items, with reference to the research of Karatzias et al. (2020), and considering the characteristics of the child participants, the focus was on family environment, interpersonal relationships, educational experiences, and physical and mental health aspects. Based on this, the scale items were correspondingly simplified to allow for easy comprehension by children, ultimately forming the 10-item scale (including 2 reverse-scored items) for children’s positive childhood experiences used in this study, such as “I have teachers who care about me.” The Cronbach’s α coefficients for the three measurements (T1, T2, T3) of positive childhood experiences were 0.914, 0.883, and 0.868, respectively, all above 0.8, indicating high reliability, stability, and homogeneity of the data. All items in this study were measured using a 5-point Likert scale, where “1” indicated “strongly disagree” and “5” indicated “strongly agree.” To facilitate children’s understanding and enhance their interest, the scale was converted into a 5-level smiley face cartoon scale.

Confirmatory factor analyses were conducted separately for the measurement models of positive childhood experiences and deviant behavior in physical education learning at T1, T2, and T3. As shown in Table 2, the measurement models for both variables demonstrated good model fit at all three time points, indicating satisfactory factorial validity of the two scales across measurement occasions. Longitudinal measurement invariance was then examined for both variables. Configural invariance (M1), metric invariance (M2), scalar invariance (M3), and strict invariance (M4) models were tested sequentially. Changes in model fit were evaluated using the criteria of ΔCFI ≤ 0.010 and ΔRMSEA ≤ 0.015. As shown in Table 3, the configural invariance model for positive childhood experiences exhibited good fit. Compared with the configural invariance model, the metric invariance model yielded ΔCFI = 0.002 and ΔRMSEA = 0.002. Compared with the metric invariance model, the scalar invariance model yielded ΔCFI = 0.001 and ΔRMSEA = 0.001. None of these changes exceeded the recommended thresholds, indicating that positive childhood experiences achieved longitudinal measurement invariance. For deviant behavior in physical education learning, all levels of invariance models also demonstrated good fit, and the changes in CFI and RMSEA for the metric, scalar, and strict invariance models remained within acceptable ranges. Overall, both variables showed satisfactory measurement stability and comparability across the three measurement occasions, providing an adequate measurement basis for the subsequent longitudinal analyses.

**TABLE 2:** Confirmatory factor analysis results at the three measurement occasions.

Variable	Time point	χ^2^	df	χ^2^/df	RMSEA 90% CI	CFI	TLI	SRMR
Positive childhood experiences	T1	82.396	32	2.575	0.074 [0.055, 0.094]	0.966	0.952	0.031
	T2	59.049	31	1.905	0.056 [0.034, 0.078]	0.975	0.964	0.032
	T3	76.591	30	2.553	0.074 [0.054, 0.095]	0.963	0.944	0.037
Deviant behavior in PE learning	T1	10.307	9	1.145	0.023 [0.000, 0.073]	0.999	0.999	0.009
	T2	22.849	9	2.539	0.074 [0.037, 0.112]	0.991	0.985	0.013
	T3	13.474	9	1.497	0.042 [0.000, 0.085]	0.996	0.994	0.015

**TABLE 3:** Results of longitudinal measurement invariance testing.

Variable	Model	ΔCFI	ΔRMSEA	χ^2^	df	CFI	TLI	RMSEA	SRMR
Positive childhood experiences	M1	−	−	556.609	361	0.952	0.942	0.044	0.044
	M2	0.002	0.002	567.86	379	0.954	0.947	0.042	0.046
	M3	0.001	0.001	587.725	397	0.953	0.949	0.041	0.047
	M4	0.015	0.005	670.859	417	0.938	0.935	0.046	0.06
Deviant behavior in PE learning	M1	−	−	130.877	114	0.996	0.995	0.023	0.028
	M2	0.003	0.006	154.524	124	0.993	0.991	0.029	0.042
	M3	0.004	0.006	179.765	133	0.989	0.988	0.035	0.045
	M4	0.002	0.002	201.254	144	0.987	0.986	0.037	0.047

M1 denotes the configural invariance model, M2 the metric invariance model, M3 the scalar invariance model, and M4 the strict invariance model with equal residual variances.

### 3.3 Procedure and methods

This study drew on established practices from previous similar research. Questionnaire assessment was conducted on a class-by-class basis using group testing methods. The survey questionnaires contained no content that interfered with children’s responses. During the completion process, the homeroom teacher was present and responsible for carefully answering students’ questions. Before the assessment, the homeroom teacher instructed students to fill out the questionnaire according to their true circumstances, assuring them that the teacher would not view their individual responses, that there were no right or wrong answers, that they would not be criticized, and that it was unrelated to academic grades. Children were free to choose whether to participate or withdraw before or during the questionnaire process if they did not wish to participate. Based on Kohlberg’s theory of reading level development (Mathes, 2021) and to ensure students fully understood the item meanings, through active communication with homeroom teachers, the test for students in grades 3, 4, and 5 was conducted by the homeroom teacher reading each item aloud uniformly. Students then considered their choices and filled in their answers directly. Only the research personnel had access to the questionnaire results. Questionnaires used digital identification, numbering participants according to school and grade. The entire test took students approximately 20 min to complete, and all questionnaires were collected on the spot.

This study employed the construction of a Latent growth model (LGM) to examine the changing trends of children’s deviant behavior in physical education learning. The latent variable growth model is a variant of structural equation modeling used to investigate the trajectory of a variable. LGM first specifies two latent growth factors, an intercept factor, and a slope factor. The intercept represents the initial status of a given variable, whereas the slope captures its trajectory of change over time. These two latent factors are subsequently estimated from the observed scores of the variable collected at multiple measurement occasions. For example, for a given individual, the observed values of a variable at three time points, t1, t2, and t3, can be expressed as a function of the latent intercept factor, the latent slope factor, and occasion-specific residuals. When the developmental trajectory is assumed to be linear, the individual growth equation can be represented in the matrix form shown below, where the two latent factors denote the initial level and linear rate of change, respectively, and the residual terms represent within-person measurement errors at the corresponding time points. Specifically, SPSS 26.0, Mplus 8.3, and Amos 23.0 were used for data analysis. The unconditional latent variable growth model and the cross-lagged regression model were analyzed using the maximum likelihood estimation method. Considering the degree of deviation from normality and the proportion of missing data in the current dataset, the final analysis retained the sample data that completed all three waves of longitudinal testing.

## 4 Results

### 4.1 Common method bias

To assess the extent of common method bias in the three measurements, Harman’s single-factor test was conducted separately on the data from each of the three testing waves. The results showed that the variance explained by the first factor was 38.281, 37.893, and 36.599%, respectively, all below the critical standard of 40%, indicating that the influence of common method bias was not significant.

### 4.2 Descriptive statistics and correlation analysis

The means, standard deviations, and correlation coefficient matrix for positive childhood experiences and children’s deviant behavior in physical education learning across the three measurements are shown in Table 4. Positive childhood experiences showed a linearly increasing trend from T1 to T3, while deviant behavior in physical education learning showed a linearly decreasing trend. The three measurements of positive childhood experiences and the three measurements of deviant behavior in physical education learning showed significant negative correlations.

**TABLE 4:** Means, standard deviations, and correlation coefficient matrix for variables.

Variable	Mean	S.D.	PCE T1	PELDB T1	PCE T2
PCE T1	3.057	0.837	1	1	1
PELDB T1	3.348	0.843	0.580**		
PCE T2	3.755	0.741	0.341**	0.423**	
PELDB T2	3.428	1.124	−0.151*	−0.229**	−0.131*
PCE T3	3.041	1.148	−0.169**	−0.252**	−0.137*
PELDB T3	2.519	0.892	−0.252**	−0.329**	−0.311**

PCE is the abbreviation for the variable Positive childhood experiences. PELDB is the abbreviation for the variable deviant behavior in physical education learning. The same applies below. T1, T2, and T3 denote the first, second, and third measurement occasions, respectively.

### 4.3 Development trajectories of positive childhood experiences and children’s deviant behavior in physical education learning

#### 4.3.1 Positive childhood experiences

A linear unconditional latent variable growth model, as shown in Figure 2, was constructed for children’s positive childhood experiences to examine the trend of change in their impact. The unconditional model demonstrated a good fit to the data (CMIN/DF for PCE and PELDB were 2.11 and 1.24, respectively, RMSEA for both were 0.06 and 0.04, SRMR for both was 0.02). The statistical results of this model are presented in Table 5. In the linear unconditional growth model, the intercept, representing the initial level of positive childhood experiences, was 3.051 (SE = 0.050, *P* < 0.001), significantly > 0; positive childhood experiences showed a linear increasing trend during the three measurement periods (slope = 0.345, SE = 0.027, *P* < 0.001). Thus, Hypothesis 1 was supported. The variance of the intercept (σ^2^ = 0.606, SE = 0.084, *P* < 0.001) and the variance of the slope (σ^2^ = 0.125, SE = 0.032, *P* < 0.001) were both significantly > 0, indicating systematic individual differences in both the initial level of positive childhood experiences and their change over time. A significant correlation existed between the intercept and slope (*r* = −0.197, *P* < 0.001), indicating that students with higher initial levels showed a smaller increase in the impact level of positive childhood experiences over time.

**FIGURE 2:**
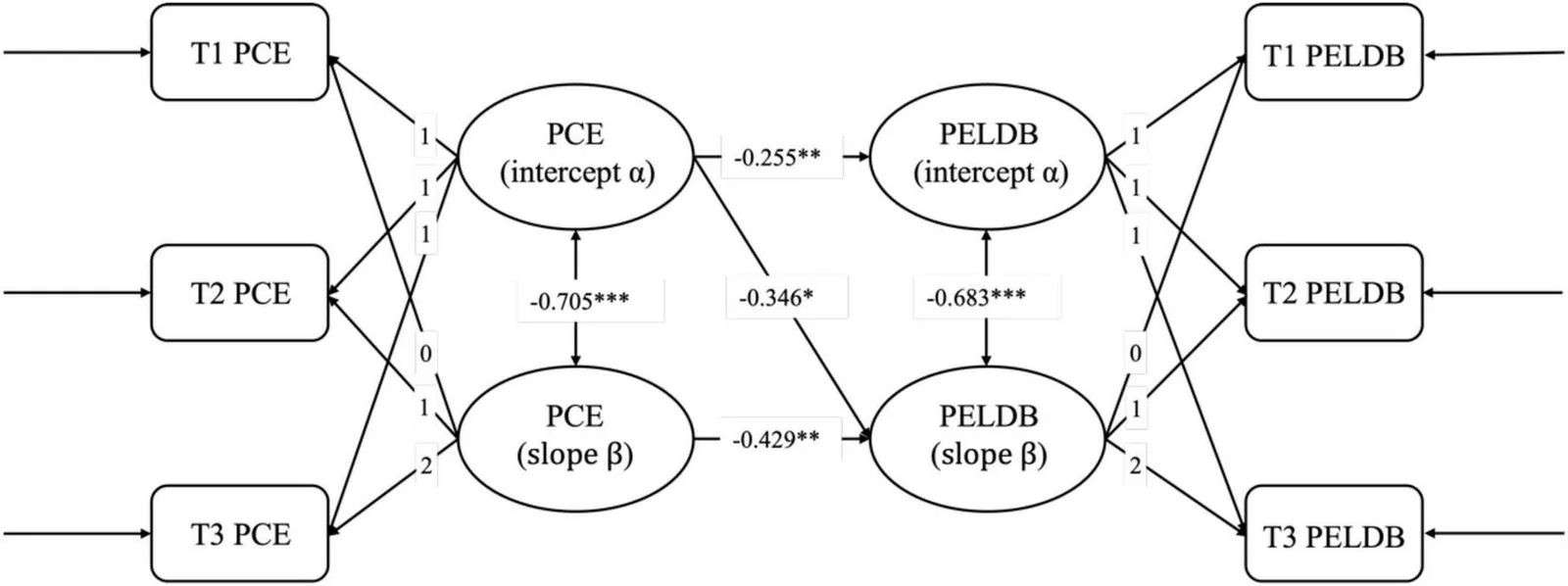
Parallel process model of positive childhood experiences and children’s deviant behavior in physical education learning.

**TABLE 5:** Estimation results of the linear unconditional LCM for PCE and DBPEL.

Model	Coefficient		Variance		Intercept-slope correlation
	Intercept	Slope	Intercept	Slope	
PCE	3.051***	0.354***	0.606***	0.125***	−0.197***
PELDB	3.445***	−0.460***	0.795***	0.206***	−0.248**

#### 4.3.2 Children’s deviant behavior in physical education learning

To examine the developmental trajectory of children’s deviant behavior in physical education learning, a linear unconditional latent variable growth model was constructed. The fit indices for this model showed CMIN/DF = 1.34 < 3, CFI = 0.99 > 0.90, TLI = 0.99 > 0.90, RMSEA = 0.04 < 0.08, SRMR = 0.02 < 0.08, indicating a good fit of the unconditional model to the data. As shown in Table 3, the model intercept, representing the initial level of deviant behavior in physical education learning, was 3.445 (SE = 0.065, *P* < 0.001), significantly > 0. Deviant behavior in physical education learning showed a linear decrease over the three measurement periods (slope = −0.460, SE = 0.036, *P* < 0.001), thus supporting Hypothesis 2. The variance of the intercept (σ^2^ = 0.795, SE = 0.145, *P* < 0.001) and the variance of the slope (σ^2^ = 0.206, SE = 0.056, *P* < 0.001) were both significantly > 0, indicating significant individual differences in both the initial level of deviant behavior and its change over time. The correlation between the intercept and slope was significant (*r* = −0.248, *P* = 0.001), indicating that children with a higher initial level of deviant behavior showed a faster decrease in their level of deviant behavior across the three surveys.

### 4.4 Parallel process model of positive childhood experiences and children’s deviant behavior in physical education learning

To test the relationship between positive childhood experiences and children’s deviant behavior in physical education learning, a parallel process model was established to simultaneously examine the latent growth of both variables. The intercept and slope from the children’s positive childhood experiences model were used to predict the linear growth of children’s deviant behavior in physical education learning. The results are shown in Figure 2. The model demonstrated an acceptable fit, with all fit indices meeting the standards (CMIN/DF = 1.64; TLI = 0.97; SRMR = 0.05).

In the parallel process latent variable model, the regression coefficient of the positive childhood experiences intercept on the deviant behavior intercept was significant (β = −0.255, SE = 0.084, *P* = 0.002), indicating that a higher initial level of positive childhood experiences was associated with a lower initial level of deviant behavior in physical education learning. Thus, Hypothesis 3 was supported. Furthermore, the positive childhood experiences intercept negatively predicted the slope of deviant behavior (β = −0.346, SE = 0.137, *P* = 0.011), indicating that a higher initial level of positive childhood experiences predicted a faster rate of decrease in deviant behavior. This result supported Hypothesis 4. Finally, the positive childhood experiences slope negatively predicted the deviant behavior slope (β = −0.429, SE = 0.140, *P* = 0.002), indicating that a faster rate of increase in positive childhood experiences over time predicted a faster rate of decrease in deviant behavior. Therefore, Hypothesis 5 was supported.

To further examine the causal relationship between children’s deviant behavior in physical education learning and their positive childhood experiences, and to check for potential reciprocal influences while avoiding potential bias from the pre-specified hypothesis, a competing parallel process model was established by swapping the independent and dependent variables. The results showed that the regression coefficient of the deviant behavior intercept on the positive childhood experiences slope was not significant (β = −0.029, SE = 0.053, *P* = 0.585). Accordingly, the present findings provide no statistically significant evidence that children’s deviant behavior in physical education learning predicted subsequent positive childhood experiences across the three measurement occasions.

### 4.5 Cross-lagged regression analysis

An increasing number of scholars suggest that multiple data analysis methods should be used comprehensively for broader sensitivity analysis to obtain more robust conclusions in causal inference (Treur et al., 2024). To further examine the lagged relationship between positive childhood experiences and children’s deviant behavior in physical education learning over time, a cross-lagged regression analysis was conducted on the three waves of data. Cross-lagged regression analysis can examine the predictive effect of one variable on another while controlling for the within-time correlations between variables and their stability across time. When using cross-lagged regression to explore causal relationships between variables, four models (as shown in Figure 3) need to be tested first: a baseline model M1 including only autoregressive effects; a hypothesized model M2 adding paths from positive childhood experiences to subsequent deviant behavior based on M1; a competing model M3 adding paths from deviant behavior to subsequent positive childhood experiences based on M1; and a full model M4 including all paths from M1, M2, and M3.

**FIGURE 3:**
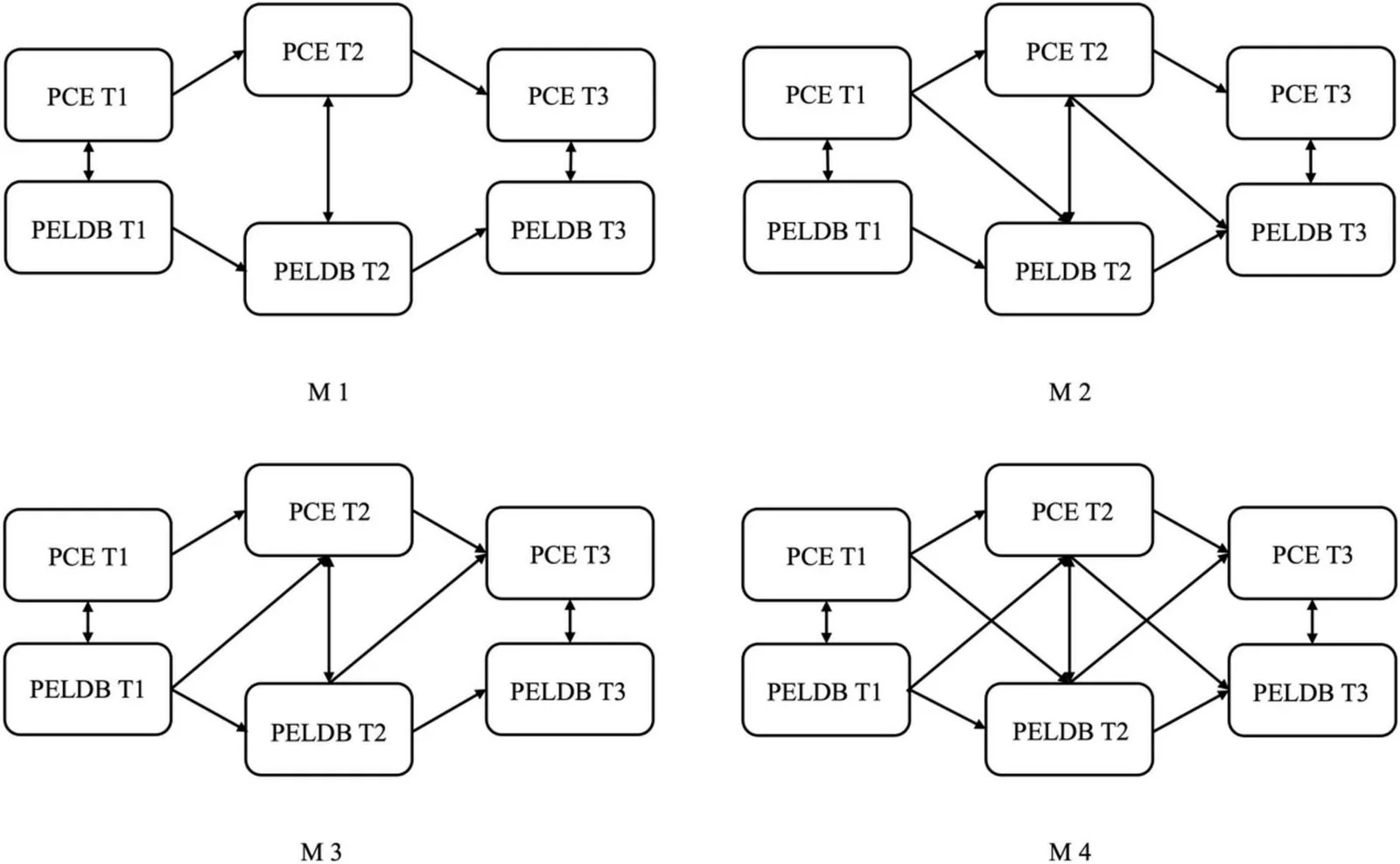
Diagram of cross-lagged regression models.

Table 6 presents the fit indices for the four models (M1–M4) and the comparison results of the Δχ^2^ for each model. The model fit indices for M2, M3, and M4 were all better than those of the autoregressive model M1. Model M3 had CMIN/DF > 3 and TLI < 0.9, indicating poor fit. Model M2 had acceptable fit indices: CMIN/DF = 2.92, CFI and TLI both > 0.9, SRMR < 0.08, RMSEA = 0.08 which is below the 0.1 threshold. The chi-square difference between M4 and M2 was significant (Δχ^2^ = 17.5, *P* < 0.01). However, analysis of the model paths revealed that the path coefficient from T2 deviant behavior to T3 positive childhood experiences in M4 was not significant (β = −0.021, SE = 0.036, *P* = 0.555). Based on these results and the principle of model parsimony, M2 was selected as the final model. The final model for positive childhood experiences and children’s deviant behavior in physical education learning is shown in Figure 4. T1 positive childhood experiences negatively predicted T2 deviant behavior (β = −0.152, SE = 0.074, *P* = 0.040), and T2 positive childhood experiences negatively predicted T3 deviant behavior (β = −0.244, SE = 0.056, *P* = 0.012). Based on this, Hypothesis 6 was supported.

**TABLE 6:** Fit indices for cross-lagged models.

Model	CMIN	DF	CFI	TLI	RMSEA	SRMR	Model comparison	Δχ ^2^	Δ df	*P*
M1	40.11	8.00	0.91	0.83	0.12	0.10				
M2	17.50	6.00	0.97	0.92	0.08	0.05	M1 vs. M2	22.61	2.00	*P* < 0.001
M3	30.33	6.00	0.93	0.83	0.12	0.08	M1 vs. M3	9.78	2.00	*P* < 0.010
M4	8.27	4.00	0.99	0.96	0.06	0.03	M1 vs. M4	31.84	4.00	*P* < 0.001

**FIGURE 4:**
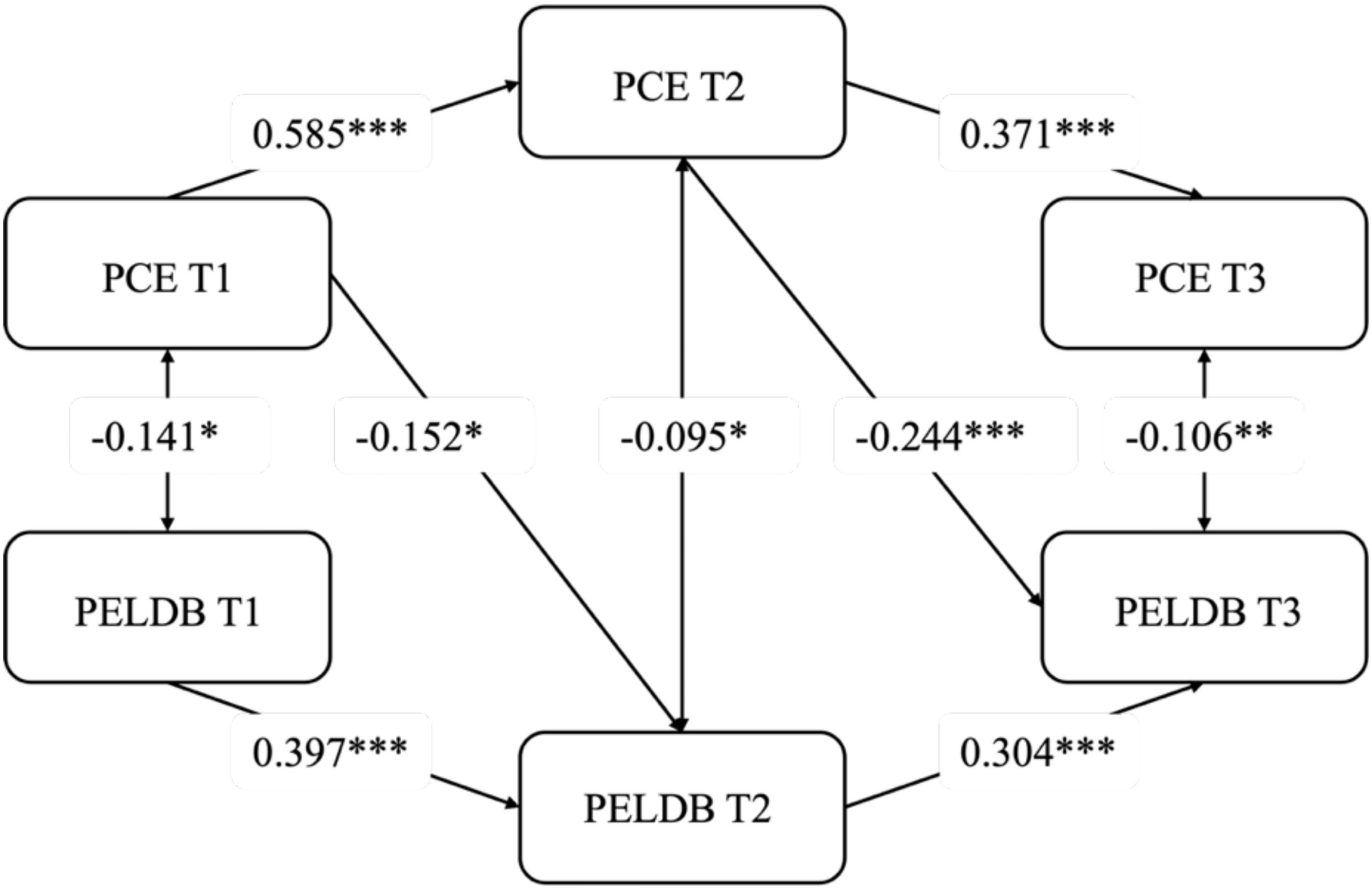
Cross-lagged regression model of positive childhood experiences and children’s deviant behavior in physical education learning.

In summary, the results from both the latent growth model and the cross-lagged regression analysis support the predictive effect of positive childhood experiences on children’s deviant behavior in physical education learning, but do not support the predictive effect of deviant behavior on positive childhood experiences. These results further indicate that the predictive effect of positive childhood experiences on children’s deviant behavior in physical education learning is a relatively robust finding.

## 5 Discussion

### 5.1 The development of the impact of positive childhood experiences on children

#### 5.1.1 The impact of positive childhood experiences on children showed a linearly increasing trend

Like the results obtained in this study, research by some scholars indicates that by age 5, children’s sensitivity to happy expressions is nearly at adult levels, but their sensitivity to other expressions gradually improves between ages 5 and 10, and even continues to improve after age 10 (Gao and Maurer, 2010). Concurrently, Cognitive Behavior Theory also emphasizes that children’s cognitive development is a process of gradual construction. From this perspective, children in early stages only have instinctive reactions to positive life experiences. As they enter the concrete operational and formal operational stages, they begin to develop more complex abstract thinking, enabling them to understand and internalize positive childhood experiences more deeply, thereby exerting a greater influence on their psychological and emotional development. The internalization process of positive childhood experiences is also a crucial aspect of child development. With increasing age, through participation in sports team activities and athletic events, the positive cooperative spirit children learn is not only an external behavioral manifestation but also becomes part of their individual value system, subsequently influencing their self-perception and attitudes toward others. Such a transformation in values does not arise automatically from participation in physical activity; rather, it is continually shaped by the specific instructional context. Accordingly, whether the values developed through physical activity can be transferred to children’s everyday lives also depends on the consistency of teacher feedback, peer norms, and educational messages across family and school settings. Only when children can repeatedly enact and reflect on relevant behaviors across different contexts can a cooperative orientation gradually evolve from a context-specific adaptive response in the classroom into a relatively stable component of their self-concept and value orientation.

#### 5.1.2 The initial level of positive childhood experiences showed a significant negative correlation with its rate of decrease

Every child is unique, and their growth paths are influenced by a combination of factors such as individual differences, family environment factors, and educational styles. Therefore, the complex phenomenon of children’s deviant behavior in physical education learning also reflects the multiple interactions in the growth process. It is well known that children with poor initial classroom performance in physical education often have greater room for improvement in subsequent learning processes. This baseline effect can be amplified by children’s self-regulatory abilities; children who see their own progress are more likely to feel encouraged and thus participate more actively in physical activities (Ng-Knight et al., 2022). Meanwhile, in the digital era where children are commonly exposed to mobile phones and the internet, they may also “precociously” construct an idealized self through labeled personas. This “performative” psychology also implicitly drives children to reduce deviant behavior in physical education.

The negative association may indicate that early experiences of belonging and success gradually diminish if they are not continually reinforced in subsequent physical education classes, particularly as peer comparison and task difficulty increase. Research on social and emotional learning in physical education suggests that self-regulation, cooperation, and relationship skills require repeated practice through embedded movement tasks, and that their joint development with physical activity is closely associated with children’s resilience and psychological wellbeing (Kalata et al., 2026). Thus, children with higher initial levels may show greater declines because of a “high-starting-point” effect, whereas those with lower initial levels may benefit more from positive feedback and emotional support (Robinson et al., 2026). Positive childhood experiences should therefore be understood as dynamic resources continually generated through classroom interaction; sustained relational support and emotional safety may be essential for slowing their decline and maintaining children’s adjustment to physical education learning.

### 5.2 The development of children’s deviant behavior in physical education learning

#### 5.2.1 Children’s deviant behavior in physical education learning showed a linearly decreasing trend

As children age, their nervous systems gradually mature, leading to significant improvements in self-control abilities and the capacity to understand complex sports rules. Their observational and imitation skills also gradually enhance. In the process of physical education learning, by observing the behaviors and performances of peers, teachers, and sports stars, children can understand and replicate positive sports behavior patterns, thereby gradually reducing deviant behaviors such as lack of concentration, violation of sports rules, and passive participation in team games. More importantly, when children exhibit positive behaviors in physical activities, they typically receive positive feedback from teachers or peers. This positive reinforcement not only strengthens children’s motivation to maintain positive behaviors but also, through feedback mechanisms, helps them recognize the value of positive behaviors, further reducing the occurrence of deviant behavior (Lufthansa et al., 2024).

Recent trauma-informed teaching research further emphasizes safety, trust, and non-punitive responses in the classroom, while viewing physical activity as a means of restoring self-regulation and relational connectedness (Subramaniam and Wuest, 2025). In physical education settings characterized by public evaluation and bodily exposure, this approach may prevent teachers from interpreting children’s resistance or impulsivity simply as disciplinary problems. Instead, it can help children gradually reduce defensive forms of deviant behavior within stable and predictable environments. Accordingly, the decline in deviant behavior in physical education learning may reflect the combined influence of individual development and supportive instructional contexts.

#### 5.2.2 The intercept and slope of children’s deviant behavior in physical education learning showed a negative correlation

Analyzing from the perspective of sports participation, children with high initial levels of deviant behavior often show lower enthusiasm in physical education classes. These children might be more inclined to find excuses to avoid participation or participate with a negative attitude. Conversely, children with low initial levels of deviant behavior are more likely to exhibit higher enthusiasm; they are more willing to try learning new sports, participate in team activities, and respond positively to teachers’ arrangements. From the perspective of sports skill learning, children with high initial levels of deviant behavior often show slower progress in skill acquisition. This phenomenon often appears in overweight children, possibly due to their lower interest in sports and higher social anxiety, such as fear of being mocked for clumsy movements, causing them to miss many learning and practice opportunities. This leads to a vicious cycle, gradually increasing their aversion to participating in physical activities. In contrast, children with low initial levels of deviant behavior, due to their positive attitude in cooperating with teachers and participating in physical activities and team games, gradually develop an increasing interest in sports. This interest becomes a significant motivator for children to participate in physical activities, enabling them to master sports skills faster and more proficiently, thereby creating a virtuous cycle that suppresses deviant behavior in physical education learning.

### 5.3 The impact of positive childhood experiences on the developmental change of children’s deviant behavior in physical education learning

#### 5.3.1 The initial level of children’s positive childhood experiences negatively predicted the initial level of their deviant behavior in physical education learning

Positive peer and teacher—student relationships, as important components of children’s positive childhood experiences, play a crucial role in fostering children’s adherence to group norms and facilitating their integration into the group. The perception of positive teacher support can reduce stress responses in the child’s amygdala, decrease cortisol secretion, and lower psychological stress levels (Hatfield and Williford, 2017). Therefore, when children experience positive social interactions during their growth, they gradually learn skills such as cooperation, sharing resources, following rules, and effective communication. However, when children lack these positive social experiences, this deficiency can limit their performance in physical education classes, affecting their learning process and outcomes, potentially leading to problems such as resistance to physical education classes and difficulties in cooperating with peers. As some studies point out, grouping children and adolescents with deviant or problematic behaviors for sports skill training might produce harmful effects (Dishion and Tipsord, 2011).

Meanwhile, a higher initial level of positive childhood experiences suggests that children enter physical education classes with greater emotional-regulation and help-seeking resources. When performing publicly, these resources may enable them to manage movement errors through emotional expression and cooperative communication rather than avoidance or resistance. Mira et al. similarly found that integrating cooperative games and relaxation exercises into physical education improved children’s emotional recovery and anxiety regulation (Mira et al., 2026). This finding suggests that social-emotional interactions in physical education can translate children’s existing positive relational resources into practical self-regulation skills.

#### 5.3.2 The lower the initial level of children’s positive childhood experiences, the faster the growth rate of their deviant behavior in physical education learning

In the process of children’s physical education learning, when children grow up in a positive and supportive environment, especially having positive experiences related to school achievement and family member recognition, they develop higher psychological resilience. A child who receives positive feedback both in life and in physical education learning will gradually build a confident mindset (Epstein, 2018). This mindset enables the child to maintain an optimistic attitude, seek solutions when facing new difficulties in physical education classes, and recover promptly from setbacks. Relatively, children who experience continuous setbacks in school, due to a lack of successful experiences and recognition, are likely to suffer a significant blow to their confidence and even self-esteem, consequently exhibiting passive or resistant behaviors in physical education learning. Over time, physical activity becomes alienated. Such a mindset not only affects their performance in physical education classes but also accelerates the growth rate of deviant behavior, intensifying the resistance to physical education.

#### 5.3.3 The rate of decrease in the impact of positive childhood experiences on children could predict the growth rate of children’s deviant behavior in physical education learning throughout the tracking period

In the process of child development, the family environment is a core component of children’s positive childhood experiences and has the most direct impact on almost all aspects, including children’s behavioral habits and emotional development (Gong et al., 2021). As Cognitive Behavior Theory emphasizes, self-efficacy is the engine for children’s action, and the family is the primary source of support for the accumulation of individual self-efficacy. The stability of the family, the quality of parent-child relationships, and the patterns of interaction among family members directly influence how children find their own positioning in the social environment, especially in collective activities like physical education learning. Regular family routines and living habits can provide psychological stability for children. Participation in family physical activities is also a marker of a healthy lifestyle. Children associate exercise with pleasant scenarios, and the subconscious connection between “fun and sports” established through emotional conditioning reflexes is more conducive to maintaining and enhancing the impact of positive life events.

#### 5.3.4 The impact of positive childhood experiences on children negatively predicted deviant behavior in physical education learning 1 year later, but the reverse was not true

In global crises such as the COVID-19 pandemic, children encountered multiple negative life events, including isolation, social restrictions, vaccinations, and various challenges. The accumulation of these events can have a certain impact on their mental health and behavioral performance, and the greater the number of risk factors children face, the higher the likelihood of them developing behavioral problems. Cognitive behavior theory emphasizes that both behavior and cognition are acquired through later learning and can be modified through positive feedback learning. Therefore, children’s positive childhood experiences can reduce the cumulative effect of negative life events, thereby lowering the risk of developing behavioral problems. Positive childhood experiences provide children with necessary emotional value and psychological resilience, helping them cope with challenges. However, this resilience cannot be built overnight but requires long-term accumulation and internalization transformation. Therefore, the impact of positive childhood experiences on children’s deviant behavior in physical education learning typically manifests after a period (Narayan et al., 2018).

From a trauma-informed teaching perspective, deviant behaviors should not be regarded as stable tendencies toward resistance or disruption in physical education; rather, they may be understood as context-dependent defensive responses that arise when children experience public evaluation or a loss of control. Consistent with cognitive behavioral theory, although such behaviors may temporarily reduce perceived threat, they are unlikely to automatically alter children’s core beliefs concerning trust and self-worth. Improvements in cognition and emotion among children exposed to complex trauma generally require structured cognitive processing and emotion regulation rather than emerging spontaneously from behavioral change alone (Morelli et al., 2025). By contrast, positive childhood experiences constitute cumulative resources that are jointly shaped over time by families, schools, and peers. Correspondingly, children’s positive adaptation involves multiple levels of influence, including child-level characteristics, family support, the school environment, and teachers’ instructional practices, and is therefore unlikely to be fundamentally transformed by a single behavioral episode in a physical education class (Bryant et al., 2025). Accordingly, although deviant behavior in physical education learning may reflect a child’s temporary decline in interest or engagement, it may be insufficient to reconstruct the child’s broader system of positive experiences within a 1-year period. This asymmetry across levels and timescales may help explain why the reverse predictive pathway was not supported.

## 6 Conclusion

This study integrated cognitive behavior theory to construct a proposed model and empirically examined the underlying relationship between positive childhood experiences and children’s deviant behavior in physical education learning. It confirmed that within the educational domain, the influence of positive childhood experiences on children increases with age, while the likelihood of exhibiting deviant behavior in physical education learning gradually decreases. Positive childhood experiences are a key factor in mitigating children’s deviant behavior in physical education learning; peer relationships, family atmosphere, teacher encouragement, and other such factors experienced during childhood are antecedent elements for reducing this deviant behavior. Children with a slower rate of decrease in the impact of positive childhood experiences also exhibited a slower rate of externalization of deviant behavior in physical education learning. The results of the cross-lagged regression analysis further supported the overall negative predictive effect of positive childhood experiences on children’s deviant behavior in physical education learning.

Based on the findings, several recommendations are proposed. First, governments should develop support systems for children’s positive development. Through policy guidance, public investment, and tax incentives, they could encourage schools and communities to organize physical activity programs centered on peer support, family connectedness, and experiences of success, such as family sports days and parent—child community events. Accessible, age-appropriate, low-cost sports spaces should also be provided to expand children’s opportunities for in-person interaction. Second, schools should replace general calls for “stronger guidance” with operational curriculum principles emphasizing low-threat participation, process-oriented feedback, peer support, and developmental assessment. Warm-ups may include non-elimination activities, such as paired coordination tasks and cooperative chasing games. Allowing children to choose task difficulty, reducing public ranking, and increasing challenges gradually may alleviate anxiety related to mistakes and peer comparison. During skill learning and group practice, teachers may form relatively stable, mixed-ability groups and rotate roles such as demonstrator, observer, encourager, and recorder, ensuring that children of different ability levels can participate and contribute. Feedback should recognize effort, progress, strategy, and cooperation rather than focus only on outcomes, while children should be allowed to retry after failure. Assessment should reduce reliance on one-off fitness scores and public competition results and include individual progress, cooperation, and emotion regulation. Schools should also strengthen teachers’ ability to identify anxiety, conflict, and early signs of deviant behavior in physical education learning through case-based training and classroom observation. Third, families should move beyond achievement- and competition-centered approaches to physical education. Through consistent companionship, communication, and shared physical activity, parents can better understand children’s difficulties in physical education. They should avoid linking motor competence or competition results to personal worth and replace criticism and comparison with encouragement and collaborative problem-solving. Ball games, outdoor exploration, and other shared activities may also be integrated into family life, enabling children to experience enjoyment, attainable success, interpersonal connection, and positive childhood experiences through physical activity.

Several limitations should be acknowledged. First, the study included only three measurement occasions, and the observation period did not cover the entire primary-school stage. Accordingly, the developmental trends identified for positive childhood experiences and deviant behavior in physical education learning should be interpreted as changes occurring within the period examined in this study. Whether these patterns remain stable across a longer developmental span requires further investigation. Second, the participants were recruited from several primary schools in western China. Given China’s large and heterogeneous population, regional differences may exist in socioeconomic conditions, school resources, the implementation of physical education curricula, and family educational environments. The present findings should therefore not be generalized directly to all primary-school children in China but should be understood primarily in relation to the sampled children and their educational contexts. Future research could recruit participants from a wider range of regions and school settings, extend the follow-up period, and include additional measurement occasions to examine the stability of the findings across different social and educational contexts and to estimate the longitudinal relationships more precisely.

## Data Availability

The original contributions presented in the study are included in the article/supplementary material, further inquiries can be directed to the corresponding author.
